# uNGAL Predictive Value for Serum Creatinine Decrease in Critically Ill Children

**DOI:** 10.3390/healthcare10081575

**Published:** 2022-08-19

**Authors:** Cristina Gavrilovici, Cristian Petru Duşa, Cosmin Teodor Mihai, Elena-Lia Spoială, Iuliana Magdalena Stârcea, Codruta Olimpiada Iliescu-Halitchi, Irina Nicoleta Zetu, Lavinia Bodescu-Amancei Ionescu, Roxana Alexandra Bogos, Elena Hanganu, Vasile Lucian Boiculese

**Affiliations:** 1 Department of Pediatrics, “Grigore T. Popa” University of Medicine and Pharmacy, 16 Universitatii Street, 700115 Iasi, Romania; 2 “Sfânta Maria” Clinical Emergency Hospital for Children, 62-64 Vasile Lupu Street, 700309 Iasi, Romania; 3 Advanced Research and Development Center for Experimental Medicine (CEMEX), “Grigore T. Popa” University of Medicine and Pharmacy, 16 Universitatii Street, 700115 Iasi, Romania; 4 Department of Orthodontics and Dento-facial Orthopedics, “Grigore T. Popa” University of Medicine and Pharmacy, 16 Universitatii Street, 700115 Iasi, Romania; 5 Departament Biomedical Science, Discipline of Rehabilitation in Pediatrics, “Grigore T. Popa” University of Medicine and Pharmacy, 16 Universitatii Street, 700115 Iasi, Romania; 6 Department of Preventive Medicine and Interdisciplinarity, Medical Informatics and Biostatistics, “Grigore T. Popa” University of Medicine and Pharmacy, 16 Universitatii Street, 700115 Iasi, Romania

**Keywords:** acute kidney injury, uNGAL, PICU, children

## Abstract

Acute kidney injury (AKI) occurs frequently in critically ill children, having an incidence of up to 26.9% and is associated with high morbidity and mortality in pediatric intensive care units (PICU). Currently, the decrease in the glomerular filtration rate is calculated using the serum creatinine levels. Nevertheless, there may be a 48 h delay between the renal injury and measurable increase in creatinine. Urinary neutrophil gelatinase-associated lipocalin (uNGAL) has been validated in relation to cardiopulmonary bypass in children, being able to detect AKI before the functional change proven by the rise in serum creatinine. Our aim was to study the utility of using uNGAL in the management of critical pediatric patients admitted to our hospital in a six month period, more specifically, its capacity to predict AKI development, alone and in the association with the renal angina index (RAI). Twenty-eight critically ill children aged from 1 day to 15 years have been included. We found that an increase in uNGAL in day 1 of admission in the PICU was significantly correlated with a decrease in creatinine clearance but not anymore in day 3. However, in our sample uNGAL did not show a significant predictability for AKI development nor the supplementary incorporation of RAI into the prediction model. Therefore, apart from cardiac surgery, the efficacy and utility or uNGAL in the management of critically ill children is still questionable. For the best prediction, we will need to incorporate not only the RAI or other PICU scores, but other biomarkers such as KIM-1, urinary cystatin, and IL 18 in larger samples.

## 1. Introduction

Acute kidney injury (AKI) occurs frequently in critically ill children, having an incidence of up to 26.9% [1] and is associated with high morbidity and mortality in pediatric intensive care units (PICU). Although not considered anymore as a true gold standard, the serum creatinine based GFR is still the most widely used appreciation of renal function. This is particularly challenging in the children population due to the variability with age, gender, and body mass [2]. The implementation of new biomarkers, among which urinary neutrophil gelatinase-associated lipocalin (uNGAL) have apparently proven their utility in predicting AKI compared with changes in creatinine clearance, in pediatric and adult studies, particularly in cardiac surgery patients [3]. Less studies have been performed in children, particularly with the aim to demonstrate the role of uNGAL in the assessment of critically ill children. 

Renal angina index (RAI), a combination of patient AKI risk and early signs of injury, was created to stratify the risk in patients for whom biomarker testing would be most optimal. 

Our aim was to study the utility of using uNGAL in the management of critical pediatric patients admitted to our hospital in a six month period, more specifically, its capacity to predict AKI development, alone and in association with the renal angina index (RAI). 

## 2. Materials and Methods

Critically ill children admitted to PICU were enrolled over 6 months (January 2021–July 2021). Inclusion criteria were a predicted discharge of >48 h of PICU admission (prediction discharge date was estimated by the attending ICU provider as part of the PICU daily clinical routine). Patients with history of end-stage renal disease, urinary tract infections, and congenital or acquired kidney disease were excluded from the study.

Data were collected at the first calendar day of PICU admission and on day 3. Urinary NGAL was assessed the day after PICU admission between 12 and 24 h after time of admission (Day 1). Day 3 was defined as the time period between 72 and 96 h after PICU admission. 

Other collected variables at the time of admission were: demographic information, admission diagnoses, comorbidities, height, weight, available laboratory values, and vital signs. Daily collected variables included vital signs, laboratory values, vasopressor use, mechanical ventilation support level, and total ICU fluid intake and output. Creatinine clearance (CrCl) (or eGFR) on day 1 was calculated according to the Schwarz formula) [(CrCl = k ∗ Ht/s.cr) k = 0.45 in infant, k = 0.55 in child]. A normal renal function was defined if the eGFR was >90 mL/min/1.73 m^2^.

All patients were also classified according to the criteria for renal angina fulfilment. The RAI score was calculated on day 1 of PICU admission. We recorded the RAI index in both ways: as a continuous variable from 1 to 40 as well as a categorical variable (dichotomous); a RAI score of ≥8 was considered as a positive index [4] We were looking for associations of uNGAL values from day 1 with CrCl from day 1 and from day 3 as well as the RAI score with the day 1 CrCl, and multiple regression RAI + NGAL as the independent and CrCl as the dependent variable.

We determined the cut-off point of uNGAL by the ROC (receiver operating characteristic) analysis to decide on a CrCl lower than 90/1.73 m^2^/min. Statistical analysis was performed using SPSS, version 18 (SPSS Inc. Released 2009. PASW Statistics for Windows, Version 18.0. Chicago). Variables were presented as the number and percent or average and standard deviations, and for some, also the 95% confidence interval. Relations between variables were assessed by means of correlation (Spearman and Pearson), and by linear or logistic regression. To compare the samples, the nonparametric Mann–Whitney test was operated. *p* ≤ 0.05 was considered significant. Multiple logistic regression analyses were performed to check the relation between independent factors (uNGAL and RAI) and CrCl > 90/1.73 m^2^/min as the output on different days. The coefficients were exponentiated to compute the adjusted odds ratio for the effect measures.

The protocol study was approved by the research ethics committees of the University of Medicine and Pharmacy “Grigore T Popa” Iasi, Romania.

## 3. Results

Twenty-eight critically ill children aged from 1 day to 15 years were included. The underlying pathology of the patients admitted in our PICU included both surgical (severe burning, diaphragmatic hernia, giant ovarian cyst, intestinal malformations, (poly)trauma, hydrocephalia, tumors (pancreatoblastoma, ovarian teratoma, Ewing sarcoma), hydropneumothorax), and medical conditions: pancreatitis, congenital cardiac malformation with cardiac failure, respiratory failure, upper gastrointestinal bleeding, adreno-genital syndrome, foreign body aspiration, and transverse myelitis. 

A total of 11/28 children (39%) had a CrCl between 22 and 87 mL/min/1.73 m^2^ on day 1 of PICU admission and 8/28 children had a CrCl between 23 and 83 mL/min/1.73 m^2^ on day 3 of PICU admission. The mean uNGAL value was 7.32 ng/mL with a large standard deviation of 10.33 ng/mL.

**Figure 1:**
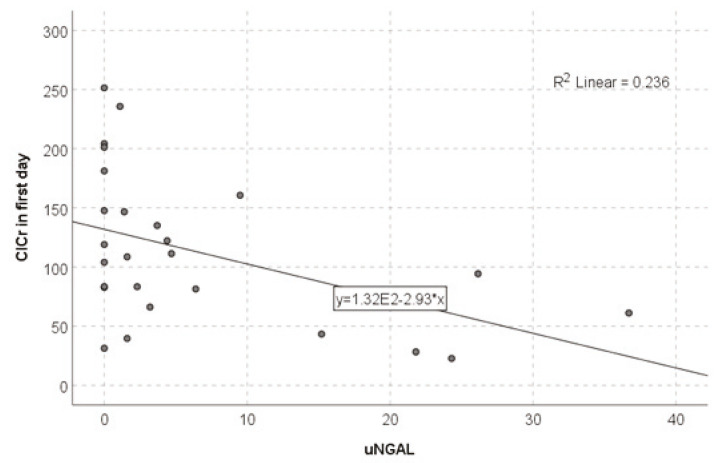
The CrCl–uNGAL relationship on day 1 of PICU admission.

We found a negative relationship between uNGAL and day 1 CrCl. The nonparametric Spearman’s rho correlation coefficient was −0.523 (with 95% CI: 0.763, 0.205, bootstrap method) and significance of *p* < 0.01. We also computed the linear trend and found a Pearson coefficient of −0.486 (with 95% CI: −0.688, −0.252) with a significance *p* < 0.05 (Figure 1, Table 1).

However, we did not find a significant relationship between uNGAL and CrCl from day 3 (Spearman’s correlation r = −0.139, *p* = 0.240) (Table 2). 

The uNGAL ROC curve analysis found a cut-off point of uNGAL = 5.55 ng/mL to predict the CrCl_1 < 90. The area under the curve (AUC = 0.708) was not statistically significant, *p* = 0.062.

**Figure 2:**
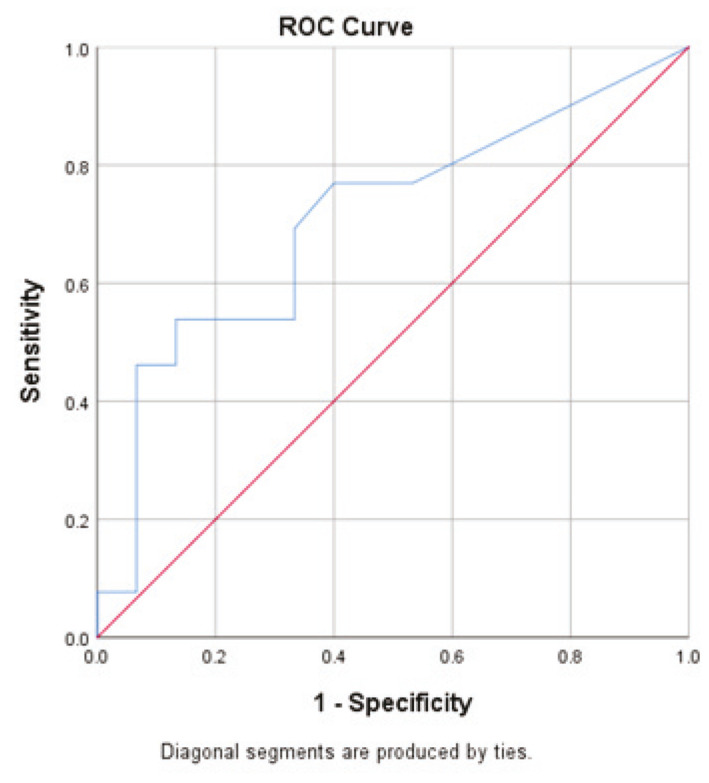
The ROC curve: uNGAL to predict the CrCl < 90 mL/1.73 m^2^/day on day 1 of PICU admission.

The corresponding sensitivity and specificity were 0.53 and 0.86, respectively (Figure 2).

The ROC analysis to predict a creatinine clearance lower than 90 on the third day, taking into account the uNGAL values, showed no significance (*p* = 0.265) and AUC = 0.643. For the optimum maximum sum of sensitivity and specificity, we found an uNGAL = 17.15. This cut-off point provided a sensitivity of 0.571 and a specificity of 0.905.

The relation between RAI and day 3 CrCl was estimated by the nonparametric Spearman correlation. The value was −0.318 with a significance *p* = 0.05. This showed a negative week relation (according to Colton classification [5]). When comparing the creatinine clearance on day 1 in the two groups (RAI positive/RAI negative), we did not find any difference (*p* = 309, Mann–Whitney U test). A similar result was obtained by comparing it with the creatinine clearance on day 3 (*p* = 0.186) (Table 3).

When assessing the relation of CrCl < 90/mL/1.73 m^2^ on the first day (as a dependent variable) in relation to the uNGAL score and a RAI larger or equal to 8, the logistic regression coefficients by means of the odds ratio (exp of beta coefficients) were 1.10 and 1.09 with a significance of 0.056 and 0.93, respectively. Therefore, there was no relation in this multiple logistic regression study. Correspondingly, we tested the same inputs in relation to the creatinine clearance on the third day. The odd ratios were 1.066 and 2.460 with a significance of 0.129 and 0.351, respectively.

## 4. Discussion

The role of uNGAL in AKI has been previously studied in small samples of children admitted to the PICU. We synthetized the main results of these studies investigating the predictive role of uNGAL (Table 4). Currently, the estimated decrease in the GFR was calculated using the serum creatinine levels. Nevertheless, there may be a 48 h delay between the renal injury and measurable increase in creatinine [5]. In the Bhowmick et al. study [6], the modified Schwartz formula showed a good agreement with the ^99m^Tc-labeled DTPA method for calculating the GFR in critically ill children aged 1 month to 12 years. Nowadays, an improved equation to estimate the GFR was developed based on Scr, BUN, and cystatin C [7]. 

uNGAL has been validated in relation to cardiopulmonary bypass in children, being able to detect AKI before the functional change proven by the rise in serum creatinine [8]. It is already known that new AKI biomarkers do not demonstrate a reliable prediction outside the cardiac surgery. However, in noncardiac PICU patients, the performance of these biomarkers is variable, with an area under the curve receiver-operating characteristic (AUC-ROC) values ranging from 0.54 to 0.85 [9]. 

We demonstrated that an increase in uNGAL on day 1 of admission in the PICU was significantly correlated with a decrease in creatinine clearance, but not anymore on day 3. Similar results were found by Dinardo et al. [9] in a smaller sample (seven patients with severe sepsis and AKI and four patients with severe sepsis and AKI) in which uNGAL was significantly increased in children with septic AKI compared with septic patients without AKI. uNGAL was not altered by sepsis, being significantly increased in children with severe sepsis + AKI compared with those without AKI. In contrast, plasma NGAL was not significantly higher in septic patients + AKI compared with septic children without AKI.

According to our ROC, uNGAL did not show a significant predictability for AKI development. A lack of uNGAL utility in AKI was demonstrated by Agarwal et al. [7] in a sample of 100 children aged 1 month to 12 years who underwent contrast-enhanced CT scan, out which 35 developed contrast induced (CI)-AKI. The NGAL did not predict contrast induced acute kidney injury while age < 2 y was an independent risk factor for CI-AKI. In studies performed on PICU patients with circulatory collapse, uNGAL has proven its efficacy in predicting AKI, with a lower performance than KIM, but better than IL18 [8]. In a much larger sample (657 children 0–16 years), McGalliard R.J. et al. [10] still did not find a good prediction of uNGAL alone. 

The renal angina index is a construct aiming for a better prediction of AKI by assigning point values for ‘risk’ (e.g., sepsis, use of vasoactives, history of transplant, and/or invasive mechanical ventilation) and ‘signs’ of injury (e.g., changes from baseline SCr, short periods of oliguria, and/or fluid overload). The resultant renal angina index score can range from 1 to 40. A cut-off of ≥8 is used to determine renal angina fulfilment [17,18].

When we supplementary incorporated the RAI into the prediction model, uNGAL + RAI did not predict better either. Unlike us, other authors [4,9,16] have demonstrated that the association of increased uNGAL + RAI ≥ 8 significantly increases the prediction for subsequent AKI. Individual uNGAL demonstrated marginal discrimination for severe AKI (AUC = 0.877, little higher than the prediction by RAI (AUC = 0.847), but the incorporation of uNGAL to the RAI significantly increased the AKI prediction (AUC = 0.847, increased to 0.893) [4]. Other authors also did not find an increased AKI prediction of such combination [19].

Our study has several limitations: the sample size was not large enough to allow us to draw definite conclusions. Although our sample included critically ill patients, the underlying disease (either medical or surgical) was not extremely severe to develop a quick AKI, nor were associated risk factors for AKI detected. Another explanation for the lack of significant results is the lack of a homogenous group. Therefore, apart from cardiac surgery, the efficacy and utility or uNGAL in the management of critically ill children is still questionable. For the best prediction, we will need to incorporate not only the RAI or other PICU scores, but other biomarkers such as KIM-1, urinary cystatin, IL-18, etc. 

## 5. Conclusions

The underestimation of GFR should be kept in mind while applying the Schwartz formula at the bedside in the PICU. Among the new biomarkers, bNGAL alone may not be useful in all PICUs. In our case, the day of admission NGAL levels were not found to be predictive of the day 3 creatinine clearance measurements. In our PICU, uNGAL did not prove its excellency in the management of critically ill children. This is also due to the small sample and the lack of homogeneity among the diseases that triggered the PICU admission. We demonstrated that uNGAL is a good marker of renal injury, but not a good predictor for AKI. Depending on the disease severity and complexity of the admitted patients, uNGAL use may not always be helpful. Indeed, to clearly demonstrate its accuracy, one may need larger samples, which is not always easy to produce, usually due to a lower frequency of such diseases in pediatrics compared to adult medicine. Last, but not least, based on ethical grounds (the reluctance of parents to accept the enrollment of children in research). However, uNGAL may be potentially helpful in reno-protective interventions such as avoiding nephrotoxic exposure and contrast agents, maintaining euvolemia, thereby decreasing the morbidity and mortality associated with AKI.

## Figures and Tables

**Table 1:** The logistic regression coefficients for the dependent variable for day 1 CrCl < 90/mL/1.73 m^2^.

Variables Involved (Type)	Correlation Confidence Interval (95%)	Significance *p*
uNGAL; CrCl day 1 (S)	−0.523 (−0.763; −0.205)	0.002
uNGAL; CrCl day 1 (P)	−0.486 (−0.688; −0.252	0.004
uNGAL; CrCl day 3 (S)	−0.139 (−0.531; 0.286)	0.24
RAI; CrCl day 3 (S)	−0.318 (−0.621; −0.073)	0.05

Spearman correlation (S), Pearson correlation (P).

**Table 2:** The logistic regression coefficients for the dependent variable for day 1 CrCl < 90/mL/1.73 m^2^.

Covariate/Factor	B	Sig. *p*	OR EXP (B)	95% Confidence Interval for EXP (B)	
				Lower	Upper
uNGAL	0.096	0.056	1.101	0.997	1.215
RAI ≥ 8	0.088	0.923	1.092	0.182	6.564

B—coefficient; OR—Odds Ratio.

**Table 3:** The logistic regression coefficients for the dependent variable for day 3 CrCl < 90/mL/1.73 m^2^.

Covariate/Factor	B	Sig. *p*	OR EXP (B)	95% Confidence Interval for EXP (B)	
				Lower	Upper
uNGAL	0.064	0.129	1.066	0.982	1.157
RAI ≥ 8	0.900	0.351	2.460	0.371	16.315

B—coefficient; OR—Odd Ratio.

**Table 4:** Relevant studies depicting the role of urinary NGAL in AKI.

Authors	Study Type	Sample Size and Age	Aims	Main Results
Abu Zeid AM et al., 2019 [10]	Prospective study	53 children, 3 months to 7 years	- To test the hypothesis that association of uNGAL and RAI improves the prediction of severe AKI.	- Individual uNGAL demonstrated marginal discrimination for severe AKI (AUC = 0.877, little higher than prediction by RAI (AUC = 0.847). Incorporation of uNGAL significantly added to the renal angina index AKI prediction (AUC = 0.847, increased to 0.893).
Acuna K.A. et al, 2018 [11]	Prospective study	34 children, 1 month–18 years	- To determine the association of uNGAL and RAI for AKI prediction in critically ill children	- Significant relationship between uNGAL and creatinine (*p* = 0.034) and no associated relationship between RAI and creatinine (*p* = 0.071). - When combined, it only showed a slight increase in the detection of AKI. (*p* = 0.067).
Agarwal Y et al, 2021 [12]	Prospective cohort study	35 children with contrast induced AKI, 1 month–12 years	- To study the diagnostic role of uNGAL and evaluate the outcome of contrast induced (CI) -AKI in critically ill children.	- There was no significant difference in NGAL 6 h after contrast-enhanced CT scan for AKI prediction (AUC 0.41, 95% CI 0.29 to 0.54) - There was no significant difference in mean plasma NGAL level before and 6 h after contrast enhanced CT scan in CI-AKI and Non-CI-AKI groups
Assadi F. et al, 2019 [13]	Prospective cross-sectional study	86 children, 7 months– 14 years.	- To assess the ability of IL-18, KIM-1), uNGAL to predict AKI in critically ill children with circulatory collapse.	- IL-18, KIM-1, and NGAL rose significantly from the day of admission to the sixth day of hospital stay (*p* < 0.001). - KIM-1 displayed the highest AUC (AUC = 0.81, 95% CI, 0.76–0.93; *p* < 0.001) for the early detection of AKI after circulatory collapse, followed by NGAL (0.77 CI, 0.70–0.84) and IL-18 (0.69, CI, 0.48–0.64)
Di Nardo M et al., 2013 [14]	Single-center prospective observational cohort study	11 children were enrolled: 7 with severe sepsis and 4 patients with severe sepsis + AKI	- As sepsis is known as a risk factor for AKI, in PICU patients, but it is also able to upregulate urinary and plasma NGAL decreasing its predicting value of AKI, this confounding factor needs to be demonstrated.	- uNGAL levels were significantly increased in patients with septic AKI compared with septic patients without AKI, while pNGAL levels were not significantly different between septic these groups.
McGalliard RJ et al., 2020 [15]	Single-center prospective, Observational cohort study	657 children 0–16 years	- To test the predictive value of uNGAL, and RAI (alone and combined) of stage 2 or) AKI development in PICU patients	This was a heterogenous PICU cohort in which, uNGAL and RAI alone did no find a good prediction for severe AKI: - The AUC for uNGAL and RAI were 0.75 (95% Confidence Interval [CI] 0.69, 0.81), and 0.73 (95% CI 0.65, 0.80) respectively. - When combined RAI + day 1 uNGAL, the AUC was 0.80 for severe AKI prediction (95% CI 0.71, 0.88).
Goldstein SL et al. [16]	Two-center prospective study	134 patients 6–18 years	- To test if a low uNGAL is a reliable tool to rule out nephrotoxic acute kidney injury in children	- uNGAL thresholds of 150 and 300 ng/mL demonstrated high specificity (92.4 and 97.1%, respectively) and negative predictive values (93.3 and 92.8%, respectively) for ruling out severe AKI.
Naunova-Timovska et al. [17]	Prospective study	50 newborns, 0–28 days	- To assess the efficiency of uNGAL in early diagnosis of AKI in newborns.	- Significant higher uNGAL values in newborn with AKI in day 1 of admission. - There was a significant higher uNGAL value in newborns with AKI and lethal outcome compared with newborns without lethal outcome (*p* < 0.001).

## Data Availability

Not applicable.
